# Cardio-Metabolic Health and HRT in Menopause: Novel Insights in Mitochondrial Biogenesis and RAAS

**DOI:** 10.2174/1573403X19666230206130205

**Published:** 2023-07-05

**Authors:** Guilherme Renke, Elaine Kemen, Priscila Scalabrin, Cleibe Braz, Thomáz Baêsso, Marcela Batista Pereira

**Affiliations:** 1 National Institute of Cardiology, Brazilian Ministry of Health, Rio de Janeiro, Brazil;; 2 Nutrindo Ideais Performance and Nutrition Research Center, Rio de Janeiro, Brazil

**Keywords:** Menopause, cardiometabolic risk factor, hormone replacement therapy, estrogen replacement therapy, mitochondria, renin-angiotensin system

## Abstract

Recent evidence shows the cardiometabolic effects of estrogen administration in postmenopausal women. Women have a cardiometabolic advantage during their reproductive years, which is lost at menopause due to declining estradiol (E2). E2, also known as 17-beta-estradiol, has diverse effects in its target tissues, including the cardiovascular (CV) system, through genomic and non-genomic signaling. Metabolic changes characteristic of menopause include a worsening lipid profile, changes in body fat distribution, epicardial and pericardial fat deposition, increased susceptibility to weight gain, and increased blood pressure, resulting in an increased risk of accelerated cardiovascular disease (CVD) development. E2 mediates its cardioprotective actions by increasing mitochondrial biogenesis, angiogenesis, and vasodilation, decreasing reactive oxygen species (ROS) and oxidative stress, and modulating the renin-angiotensin-aldosterone system (RAAS). In this review, we assess whether it is prudent to develop an approach to managing postmenopausal women based on modifying the patient's CV risk that includes human-identical hormone replacement therapy (HRT), modulation of RAAS, and stimulating mitochondrial biogenesis. Further research is needed to assess the safety and benefit of HRT to reduce cardiometabolic risk.

## 1 INTRODUCTION

The leading cause of death worldwide is ischemic heart disease, responsible for 16% of all deaths worldwide. Since 2000, the most significant increase in deaths has been due to this disease, rising from more than 2 million to 8.9 million deaths in 2019, including postmenopausal women [1].

Women have a cardiometabolic advantage during the reproductive years, and this is lost at menopause, a period marked by ovarian senescence and a drastic decline in estradiol (E2) levels [2]. The end of a woman's reproductive life coincides with increased susceptibility to cardiovascular disease (CVD), suggesting that ovarian hormone deficiency may play a critical role in the development of CVD [3].

The metabolic changes characteristic of menopause include worsening lipid profile, changes in body fat distribution, epicardial and pericardial fat deposition, increased susceptibility to weight gain, decreased insulin sensitivity, and increased blood pressure, resulting in an increased risk of accelerated development of CVD [4].

E2, also known as 17-beta-estradiol, is the most abundant form of estrogen circulating in the human body and is considered the primary female hormone [2]. Two other natural forms occur in less abundance, namely, estrone (E1) and estriol (E3) [2, 3]. Estrogens have several effects on their target tissues, including those of the cardiovascular system (Table **1**), when involved in a complex interplay of genomic and non-genomic signaling events mediated by estrogen receptors (ER). The genomic effects of estrogens are mediated by their interaction with nuclear estrogen receptors alpha (ERα) and beta (ERβ), additionally, their non-genomic effects occur *via* membrane ER, especially the G protein-coupled estrogen receptor (GPER) [3].

The rapid effects of estrogen on the blood vessel wall are thought to occur without any change in gene expression (non-genomic effects), such as arterial vasodilation in response to estrogen administration, while the long-term effects involve changes in gene expression (genomic effects) [4, 5].

Estrogen alters serum lipid concentrations, coagulation and fibrinolytic systems, antioxidant systems, and the production of other vasoactive molecules, such as nitric oxide (NO) and prostaglandins, which may influence the development of CVD [6].

In endothelial and vascular smooth muscle cells, estrogens, especially E2, bind to ERα, Erβ, and GPER receptors and activate a cascade of intracellular signaling pathways, releasing NO, vascular relaxation, and vasodilation [7]. They can cause a reduction in the production of vasoconstrictors, such as endothelin and angiotensin II. ERα and ERβ were found in male and female cardiomyocytes [6, 8]. However, one study showed transcripts only for ERα in cardiomyocytes isolated from neonatal or adult rats, suggesting that ERβ could be present in other types of cardiac cells, such as fibroblasts or vascular cells [9].

In addition to their presence in the nuclei and plasma membrane, ERα and ERβ have been detected in the mitochondria of many cell types and species [8, 9]. However, ERβ appears to be the main ER present in mitochondria, as demonstrated by immunohistochemistry, immunocytochemistry, and immunoblots using a large panel of antibodies, and import mechanisms have been studied [9]. In this review, we evaluated the possible benefits of HRT for CV and metabolic health through improved mitochondrial metabolism and reduced RAAS activity.

## 2 ESTROGEN AND MITOCHONDRIAL BIOGENESIS

The effects of estrogens on mitochondria are not restricted to their presence and role within mitochondria. The expression of mitochondrial proteins is mainly controlled by the nuclear genome, so the nucleus controls mitochondrial biogenesis [9]. It is, therefore, not surprising that estrogen's primary effects on mitochondria stem from its nuclear effects. Estrogens play a protective role in mitochondria through direct and indirect effects on various tissues [10, 11].

Estrogen regulation of mitochondrial mass and function has been shown to participate in vascular and cardiac protection [12-15]. Estrogens modulate several aspects of mitochondrial function, including ATP production, ROS generation, antioxidant defenses, mitochondrial membrane potential, and calcium manipulation. Furthermore, estrogens *via* ERs are involved in the mitochondrial life cycle controlling mitochondrial biogenesis, quality control, and mitophagy. These effects can be mediated by genomic and non-genomic effects.

Estrogens favor mitochondrial biogenesis in a tissue-specific manner by stimulating or inhibiting the expression of mitochondrial proteins from the nuclear and mitochondrial genomes [10, 11]. Estrogens directly modulate the expression of mitochondrial protein genes by binding ER/E2 to the ER of metabolic genes in the nucleus. They increase the expression of the master regulator of energy metabolism and mitochondrial biogenesis of Peroxisome proliferator-activated receptor gamma coactivator 1-alpha (PGC-1α) and its downstream targets [10, 16]. E2 also stimulates mitochondrial biogenesis in different cardiac cells such as cardiomyocytes, endothelial cells, and cardiac fibroblasts by ER/E2-mediated activation of transcription factors [12]. E2 also blocks the activation of c-Jun N-terminal kinase (JNK), which has been related to the development of mitochondrial dysfunction [16, 17]. Nuclear respiratory factor 1 (NRF1) increases the transcription of genes encoded in the mitochondrial nucleus by increasing the production of the mitochondrial transcription factor A (mtTFA) [18]. This is mediated by ERα and the presence of E2 in the promoter of NRF1 that can bind to both ERα and ERβ in an estrogen-dependent manner [17].

In addition, estrogens reduce inflammation and the secretion of proatherogenic cytokines such as tumor necrosis factor-alpha (TNF-α) and may increase prostaglandins, which reduce oxidative stress and platelet activation [19]. As discussed above, they also have numerous beneficial effects on mitochondria, including increased transcription of mitochondrial genes and increased activation of proteins involved in ROS removal [20].

## 3 ESTROGEN AND RENIN-ANGIOTENSIN-ALDOSTERONE SYSTEM (RAAS)

The RAAS is an important immunomodulating system with crucial regulatory roles which consists of two different counterbalancing pathways. One proinflammatory and the other with anti-inflammatory properties. Both pathways are vital to the regulation of renal and CV systems, regulating blood pressure and electrolyte balance [2]. In the event of trauma or exposure to an infectious agent, RAAS is also responsible for an accurate, targeted, and resolute immune response. An imbalance in RAAS causes an upregulation in its proinflammatory pathway contributing to the development of cardiac and endothelial dysfunction [2].

Estrogen plays an important role in the conversion system of RAAS [21, 22]. The RAAS is an immunomodulatory system that is critical to maintaining homeostasis in various systems. Recognizing the critical need to balance the pro-inflammatory and anti-inflammatory arms of the RAAS facilitates an appreciation of the myriad ways in which the E2 works to modulate RAAS. The various interactions between E2 and RAAS help to maintain cardiometabolic homeostasis [2]. Both immune and cardiometabolic function decline with reduced E2 production, in part, because the RAAS becomes dysregulated by E2 deficiency, leaving the RAAS predominantly in its pro-inflammatory state and predisposing it to low-grade systemic inflammation [2, 21]. E2 restores the vasodilatory effect, normalizes receptor functions and nitric oxide levels, and reduces the production of ROS [21].

E2 deficiency and RAAS dysregulation contribute to impaired immune responses, oxidative stress, and an increased incidence of cardiac hypertrophy, hypertension, atherosclerotic cardiovascular disease, arrhythmias, and heart failure [2]. The increased risk of CVD appears to be related, in part, to the loss of protection offered by endogenous steroid hormones [21]. E2 is a signaling agent that plays an important role in determining which RAAS pathway predominates [2]. It is a hormone known as an anti-inflammatory agent that inhibits the production of inflammatory cytokines such as IL-6, TNFα, and interleukin 1β (IL-1β). The increase in CVD may be due to a lack of E2. The possible mechanism for these changes is the increase in angiotensin II and the loss of inflammatory balance in favor of postmenopausal inflammatory cytokines [22].

E2 influences the activation of the “switch” to restore the anti-inflammatory state. Estrogen, in the dominant form produced by ovaries of reproductive age, E2, is protective of cardiovascular structures through its immunomodulatory effects on the renin-angiotensin-aldosterone system [2, 21, 22]. This gives women of childbearing age the “health advantage” they have until menopause, but after menopause, the loss of E2 increases the CV risk. Based on current data showing long-term safety with using human-identical HRT as a preventive step to prevent the inevitable development of a dysregulated RAAS [22].

## 4 ESTROGEN REPLACEMENT THERAPY AND CVD

Although abundant data support E2 as a cardioprotective agent in experimental CVD models, E2 replacement therapy in postmenopausal women has been very controversial. In the 1990s, several double-blind, controlled studies of postmenopausal estrogen replacement, including the Women's Health Initiative (WHI) and the Heart and Estrogen/progestin Replacement Study (HERS), were conducted [23, 24].

The failure of both trials to support the protective role of HRT in reducing CVD may, in part, be due to the administration of HRT long after the onset of menopause in the majority of women enrolled. A new hypothesis, the “critical window of hormone therapy”, has recently received much attention. The hypothesis supports the view that HRT can be effective if started early at the onset of menopause, such that late postmenopausal estrogen replacement occurs in a tissue state markedly different from 10 years earlier when estrogen production ceased [25].

Unlike the HERS and WHI studies, the Kronos Early Estrogen Prevention Stud (KEEPS) and Early *versus* Late Intervention Trial with Estradiol (ELITE) studies support the timing hypothesis and do not suggest any significant harm with possible CVD benefits of initiating HRT at the time of menopause [3, 26].

The ELITE study concluded that estradiol therapy was associated with less progression of subclinical atherosclerosis than placebo when therapy was started within 6 years of menopause, but not when therapy was started 10 or more years after menopause [26].

The KEEPS study showed that HRT improved mood, sleep, vasomotor symptoms, and bone density without increasing CV adverse events, suggesting that there may be non-cardiac benefits and safety in using HRT in recently postmenopausal women [3].

Patients with vasomotor symptoms aged <60 years and/or within 10 years of onset of menopause and healthy individuals without contraindications [prior stroke, acute myocardial infarction (AMI) or pulmonary embolism (PE), high-risk breast cancer or venous thromboembolism (DVT)] are candidates for HRT. In women who may benefit from HRT's vasomotor, genitourinary, and bone health properties, CVD risk should be individualized and optimized before initiating therapy [19, 27].

Estrogen mediates its cardioprotective actions by managing RAAS, increasing angiogenesis and vasodilation, decreasing ROS, and oxidative stress fibrosis. E2 limits cardiac remodeling through these mechanisms and attenuates cardiac hypertrophy [27]. However, menopausal women lose the cardiometabolic advantage conferred by estrogen when they experience a drastic drop in E2. When physiological doses of identical human hormones are used in HRT, accompanied by an optimal lifestyle, postmenopausal women have the most potential to achieve healthy longevity [26, 27]. Recent evidence suggests that, when initiated within 10 years of menopause, HRT reduces all-cause mortality and risks of coronary disease, osteoporosis, and dementia. [24-27].

## CONCLUSION

Although HRT is not currently recommended for primary or secondary prevention of CVD for women at high risk of CVD, in selected populations, it can be very beneficial due to its cardiovascular, immunomodulatory, and antioxidant effects. Attention to the critical window of hormone therapy and the route of administration, dose, and combination of E2 with progestin are essential factors to be individualized. Human-identical HRT could be an excellent ally for women when properly used, especially in modulating RAAS and stimulating mitochondrial biogenesis, thus reducing cardiometabolic risk. Further research is needed to assess the safety and benefit, indicating that HRT reduces CV risk.

## Figures and Tables

**Table 1:** Multiple cardiovascular effects of Estradiol: endothelial function, mitochondrial metabolism, immunomodulation, and RAAS.

	**References**
Reduces serum lipid concentrations, stabilizes coagulation and fibrinolytic systems, activates antioxidant systems and prevent the development of CVD.	[6]
Stimulates the release of NO, favoring vascular relaxation and vasodilation.	[7]
Favors cardiovascular protection by increasing mitochondrial biogenesis.	[11-16]
Reduce inflammation, oxidative stress, and platelet activation.	[20]
Increase the transcription of mitochondrial genes and activation of proteins involved in ROS removal.	[21]
Restores the vasodilatory effect, normalizes receptor functions and nitric oxide levels, and reduces the production of ROS.	[23]
Protects cardiovascular structures through its immunomodulatory effects on the renin-angiotensin-aldosterone system (RAAS).	[22, 24]
Attenuates cardiac hypertrophy and remodeling.	[30]

## LIST OF ABBREVIATIONS

AMI: Acute Myocardial Infarction
CV: Cardiovascular
CVD: Cardiovascular Disease
DVT: Venous Thromboembolism
E1: Estrone
E2: Estradiol
E3: Estriol
ELITE: Early *versus* Late Intervention Trial with Estradiol
ER: Estrogen Receptor
ERα: Estrogen Receptor Alpha
ERβ: Estrogen Receptor Beta
GPER: G Protein-Coupled Estrogen Receptor
HERS: Estrogen/progestin Replacement Study
HRT: Hormone Replacement Therapy
JNK: c-Jun N-Terminal Kinase
KEEPS: Kronos Early Estrogen Prevention Stud
mtTFA: Mitochondrial Transcription Factor A
NO: Nitric Oxide
NRF1: Nuclear Respiratory Factor 1
PE: Pulmonary Embolism
PGC-1α: Peroxisome Proliferator-Activated Receptor Gamma Coactivator 1-alpha
RAAS: Renin-Angiotensin-Aldosterone System
ROS: Radical Oxygen Species
TNF-α: Tumor Necrosis Factor Alpha
WHI: Women's Health Initiative
